# Innovations in Chest Imaging: How Can Patients Benefit?

**DOI:** 10.3390/diagnostics14020141

**Published:** 2024-01-08

**Authors:** Christian Booz

**Affiliations:** Division of Experimental Imaging, Department of Diagnostic and Interventional Radiology, University Hospital Frankfurt, 60590 Frankfurt, Germany; boozchristian@gmail.com

This Special Issue of *Diagnostics* entitled “Leading Diagnosis on Chest Imaging” provides an overview of recent technical developments in chest imaging and their clinical relevance, with a special focus on dual-energy CT (DECT) imaging.

The increasing use of DECT in clinical practice has allowed for multiple applications of reconstruction algorithms to be explored, thanks to material decomposition, to obtain more information about the composition of tissues or to increase the contrast through virtual monoenergetic images (VMIs), potentially reducing the amount of contrast agent given to patients. In this Special Issue, a review provides an overview of DECT’s technical principles of emission- and detector-based platforms and of the available post-processing algorithms (such as VMI), iodine maps, virtual non-contrast imaging (VNC), and virtual non-calcium (VNCa), focusing on their clinical applications in cardiothoracic studies. The use of VMI algorithms has proven to be particularly valuable in cardiac imaging. In this context, an original article in this Special Issue on VMI in coronary computed tomography angiography (CCTA) showed how 40 keV VMI datasets provided the highest objective and subjective image quality while maintaining excellent correlation with conventional images for the detection and grading of coronary artery stenosis. In addition, there is a case of a 65-year-old severely obese woman affected by severe aortic valve stenosis who underwent a TAVI procedure that was complicated by the embolization of the device. The patient underwent spectral CT angiography that allowed for improved image quality by means of VMI reconstructions, enabling proper pre-procedural planning. In another case report in this Special Issue, the authors highlight how DECT-derived VMI series and iodine maps assisted the prompt detection of anterior wall infarction due to LAD occlusion in an 80-year-old patient, which was not detectable in conventional images, allowing for early treatment and patient survival.

Another application of DECT is presented in a case report, describing the case of a 17-year-old female with a history of tracheoesophageal fistula who underwent DECT chest angiography. The images show the presence of high-density foci in the right lung and spectral. A spectral analysis with color-coded Z-effective maps revealed the presence of a material with a higher atomic number than iodine, enabling the diagnosis of barium pulmonary granulomas.

Research efforts in recent years have also focused heavily on SARS-CoV-2 infection, which had an intense impact on public health as a result of its global spread and high morbidity and mortality. Imaging played a key role in both the diagnosis and assessment of the degree of severity of the infection. In this context, the authors evaluated the predictive values of quantitative CT indices of the total lung and lung lobe tissue at discharge for the pulmonary diffusion function of patients affected by COVID-19 infection 5 months after symptom onset. The authors found the well-aerated lung and mean lung density to be potential indicators of diffusion function. In another original article in this Special Issue, the authors searched for risk factors that may predict the devolvement of a pneumothorax (PNX) in COVID-19 patients, showing that advanced age and male gender increase the probability of PNX.

Technological innovation also led to the introduction of new systems that may support chest imaging. In this context, a convolutional neural network (CNN) model called LW-CORONet was evaluated in this Special Issue, requiring fewer parameters and less memory space compared to state-of-the-art deep learning algorithms in order to facilitate the detection of typical COVID-19 features in chest X-ray images, which can support radiologists in the diagnostic process. In addition, a three-dimensional virtual reality tool for the visualization and localization of lung tumors from chest CT datasets was developed and evaluated in this Special Issue, integrated with a wearable thermo-haptic device providing thermal feedback, which was supposed to provide a better comprehension and characterization of tumors. The technology was tested by a medical team who reported encouraging results.

The spread of DECT technology and its increasing availability at different institutions has enormous potential, both in scientific and clinical contexts; however, it is important to ensure that the applications and limitations of this technology are adequately deployed, which was one of the main goals of this Special Issue. Specifically, this topic has been extensively covered thanks to reviews, original articles, and case reports that highlight the different applications of spectral CT algorithms. Importantly, the new photon-counting CT (PCCT) technology provides the possibility to obtain not only intrinsic spectral data but also ultra-high-resolution images, further increasing the diagnostic performance while allowing for a lower radiation dose to be used, especially in the lungs and heart. PCCT has overcome the restrictions of conventional CT spatial resolution in visualizing lung structures and quantifying coronary calcified plaques. Surely, further studies will be needed to validate this type of technology [1,2].

Artificial intelligence is also arousing considerable interest in the scientific world, especially in radiology. Many studies have been conducted so far on the possible applications of AI in the cardiothoracic field, ranging from the segmentation of lesions to automatic classification, the improvement of image quality, and support in the diagnostic analytic process [3]. During the COVID-19 pandemic, numerous AI models were developed and published for diagnosis support and severity prediction; this Special Issue presents an example of one of these tools. However, validation studies will be necessary to estimate the clinical impact of AI algorithms.

Unquestionably, the use of these software is directing the future of imaging towards greater reproducibility, reducing the inter-individual variability in the interpretation of images, and it is shifting the focus towards increasingly quantitative parameters, not only through AI, but also through the advent of radiomics. However, the development of radiomics is a particularly slow process which must deal with several problems, such as the identification of features that correlate with a certain pathology or with its behavior and also the correct recognition of such elements within images [4].

I am confident that this volume can have a major scientific impact, as it provides knowledge about recently developed innovations to improve chest imaging. It presents a fine selection of different postprocessing and analysis tools, and finally shows how patients can benefit from these technical developments in clinical routines.

## Data Availability

No data are available in the context of this editorial.
